# RNA-seq analysis identifies transcriptomic profiles associated with anal cancer recurrence among people living with HIV

**DOI:** 10.1080/07853890.2023.2199366

**Published:** 2023-05-13

**Authors:** Yuanfan Ye, Kevin J. Maroney, Howard W. Wiener, Olga A. Mamaeva, Anna D. Junkins, Greer A. Burkholder, Staci L. Sudenga, Mohd Khushman, Sameer Al Diffalha, Anju Bansal, Sadeep Shrestha

**Affiliations:** a Department of Epidemiology, School of Public Health, University of Alabama at Birmingham, AL, USA; b Department of Medicine, Division of Infectious Diseases, School of Medicine, University of Alabama at Birmingham, Birmingham, AL, USA; c Division of Epidemiology, Vanderbilt University Medical Center, Nashville, TN, USA; d O’Neal Comprehensive Cancer Center, University of Alabama at Birmingham, Birmingham, AL, USA; e Department of Pathology, School of Medicine, University of Alabama at Birmingham, Birmingham, AL, USA

**Keywords:** RNA-seq, anal cancer, treatment response, PLWH, transcriptome profile, cancer recurrence

## Abstract

**Background:**

Chemoradiation therapy (CRT) is the standard of care for squamous cell carcinoma of the anus (SCCA), the most common type of anal cancer. However, approximately one fourth of patients still relapse after CRT.

**Methods:**

We used RNA-sequencing technology to characterize coding and non-coding transcripts in tumor tissues from CRT-treated SCCA patients and compare them between 9 non-recurrent and 3 recurrent cases. RNA was extracted from FFPE tissues. Library preparations for RNA-sequencing were created using SMARTer Stranded Total RNA-Seq Kit. All libraries were pooled and sequenced on a NovaSeq 6000. Function and pathway enrichment analysis was performed with Metascape and enrichment of gene ontology (GO) was performed with Gene Set Enrichment Analysis (GSEA).

**Results:**

There were 449 differentially expressed genes (DEGs) observed (390 mRNA, 12 miRNA, 17 lincRNA and 18 snRNA) between the two groups. We identified a core of upregulated genes (*IL4, CD40LG*, *ICAM2*, *HLA-I (HLA-A, HLA-C)* and *HLA-II (HLA-DQA1, HLA-DRB5)* in the non-recurrent SCCA tissue enriching to the gene ontology term ‘allograft rejection’, which suggests a CD4+ T cell driven immune response. Conversely, in the recurrent tissues, keratin (*KRT1, 10, 12, 20*) and hedgehog signaling pathway (*PTCH2*) genes involved in ‘Epidermis Development,’, were significantly upregulated. We identified miR-4316, that inhibit tumor proliferation and migration by repressing vascular endothelial growth factors, as being upregulated in non-recurrent SCCA. On the contrary, *lncRNA-SOX21-AS1*, implicated in the progression of many other cancers, was also found to be more common in our recurrent compared to non-recurrent SCCA.

**Conclusions:**

Our study identified key host factors which may drive the recurrence of SCCA and warrants further studies to understand the mechanism and evaluate their potential use in personalized treatment.

**Key Message**
- Our study used RNA sequencing (RNA-seq) to identify pivotal factors in coding and non-coding transcripts which differentiate between patients at risk for recurrent anal cancer after treatment. There were 449 differentially expressed genes (390 mRNA, 12 miRNA, 17 lincRNA and 18 snRNA) between 9 non-recurrent and 3 recurrent squamous cell carcinoma of anus (SCCA) tissues. The enrichment of genes related to allograft rejection was observed in the non-recurrent SCCA tissues, while the enrichment of genes related to epidermis development was positively linked with recurrent SCCA tissues.

## Introduction

Anal cancer comprises 1.5% of gastrointestinal malignancies [1]. Although multiple tumor types can arise in the anal canal, squamous cell carcinoma of the anus (SCCA) is the most common histologic diagnosis (85%) and is often referred to as ‘anal cancer (AC)’. It mainly occurs in older adults in the general population, with an average age at diagnosis in the early 60s [2]. In 2019, there were an estimated 74,752 people living with anal cancer in the US. In 2022, an estimated 9400 new cases will be diagnosed, along with approximately 1670 deaths nationwide [3]. According to the most recent surveillance report by the National Cancer Institute, the overall incidence increased by 4.6% per year from 2001 to 2009, with no significant reduction from 2009 to 2015 [4]. The risk of SCCA is 24-fold higher in women living with HIV and >50-fold higher in men living with HIV (MLWH) compared to the general population [5–8]. The incidence rates of SCCA are 129-fold higher in men who have sex with men (MSM) with HIV, compared to the general US population [9]. It is the second most common non-AIDS-defining malignancy and a leading cause of morbidity among people living with HIV (PLWH) in the US.

Chemoradiation therapy (CRT), the standard treatment for SCCA [10–12], has shown a superior treatment rate compared to other treatments. However, 10–26% of patients do not have a complete response to CRT, and about 24% of patients may relapse [13–16]. Further, treatment outcomes for PLWH are not as well described as for individuals living without HIV. Given the limited number of SCCA cases, a conventional clinical trial is limited and molecular approaches have been explored. Yanik et al. [17] indicated that there were no differences with immune-related expression profiles or check-point molecule expression (e.g. PD-L1) in SCCA by HIV status. However, Yanik’s study combined tumor tissues from treated and untreated individuals and importantly only select candidate immune-related genes were examined in 3 PLWH and 4 patients without HIV. A multi-platform profiling analysis of SCCA tissues by Smaglo et al. suggested over-expression of proteins involved in resistance to chemotherapeutic drugs such as excision repair cross-complementing gene 1 (*ERCC1*) and multi-drug resistance-associated protein 1 (*MRP1*) that offered therapeutic investigation [18]. Other genomic profiling studies suggested no unique patterns between primary SCCA and recurrent SCCA [19]; mutations in *PIK3CA, FBXW7, MLL2*, and *MLL3* appeared to be common among primary and metastatic SCCA [20–23]. However, to date there are no biological signatures that determine CRT response in SCCA patients.

Identifying predictive biomarkers regarding therapeutic impacts and disease prognosis using RNA-sequencing (RNA-seq) based approaches has been widely applied in the field of cancer research [24]. While the use of this approach revealed significant coding and non-coding transcripts in various cancers [25], transcriptomic signatures in SCCA are lacking, specifically with treatment response outcomes. In this study, we characterized gene expression profiling in SCCA tumor tissues from PLWH to compare coding (mRNA) and non-coding (e.g. miRNA, IncRNA) transcripts that were differentially expressed among successful CRT versus recurrent SCCA patients. The transcriptomic biomarkers identified in this study can provide insights into the pathological processes driving observed treatment outcomes and need to be validated in larger cohorts in future studies to determine whether they can be accurately used to screen for early diagnosis of patients at high risk of having recurrent anal cancer.

## Methods

### Cohort and study design

Electronic health records (EHR) of PLWH attending the University of Alabama at Birmingham (UAB) HIV Clinic (the 1917 Clinic) between 1 January 2006 and 31 March 2018 were reviewed. Each patient was retrospectively reviewed 5 years after the cancer diagnosis or until the end of the most recent clinical visit. In accordance with standard procedures at the UAB Comprehensive Cancer Center (UAB-CCC), all SCCA (ICD-O-3 site code C20.9, C21.0–C21.9) are recorded in the EHR. These were reviewed by an oncologist and the linked cancer tissues in the biorepositories were microscopically confirmed and validated by a pathologist. Non-recurrence is defined as the absence of disease at the site of the primary tumor and regional lymph nodes within 6 months from the end of chemoradiation therapy. Local recurrence (LR) is defined as persistent disease or recurrence at the site of the primary tumor. Each patient’s EHR was retrospectively reviewed up to 5 years after the last session of chemoradiation therapy to determine if LR occurred. Tumor tissues linked with the confirmed SCCA diagnoses were requested from the UAB Tissue Biorepository.

### RNA extraction

RNA was extracted from unstained formalin-fixed, paraffin-embedded (FFPE) slides. We obtained five 5 mm-thick unstained FFPE slides of each patient (9 CR and 3 LR). The tissues from five FFPE sections were combined and used for the simultaneous isolation of total RNA and DNA with Quick-DNA/RNA FFPE kit (R1009) from Zymo Research, Costa Mesa, CA according to the manufacturer’s protocol. RNA was treated with DNase1 in the columns. The concentration and purity of RNA were assessed using Nanodrop spectrophotometer (Thermo Scientific, Waltham, MA). Additional QA/QC of the samples was conducted in the Genomics Core Laboratory at the University of Minnesota using Agilent BioAnalyzer/TapeStation Analysis. Assays were performed with 20 ul volume, with a minimal of total 400 ng of RNA.

### Library preparation and RNA-sequencing

Libraries were created using SMARTer Stranded Total RNA-Seq Kit v2 – Pico Input Mammalian (Takara Bio USA, Inc, San Jose, CA), as described in the manufacturer’s technical note (https://www.takarabio.com/Pdf/RenderPdf). All libraries were pooled and sequenced on a NovaSeq 6000 (Illumina, San Diego, CA). Depths of >40 million paired-end 150 bp reads were generated for each sample and all expected barcodes were detected. The overall sequencing quality among all libraries was consistently good, with an overall average mean quality score of >Q30.

### Bioinformatics analysis

Raw FASTQ sequences were first trimmed to remove adapter sequences to a minimum sequence length of 75 bp with a mean Phred score cutoff of 30 to ensure only high-quality reads were used for alignment and counting using Trim Galore as previously described [26,27]. Input RNA-seq reads were aligned to the GRCh38 human reference genome using HISAT2 software [28–31]. Transcript abundance was measured using featureCounts and the matrix of counts served as the unit for the differentially expressed gene (DEG), which was conducted using the bioconductor R package, edgeR [32]. Utilizing the ‘summarizeOverlaps (mode = Union)’ function, only those reads that overlapped with at least one feature, as well as overlapped between paired-end reads were counted. A combination of negative binomial distribution, Bayes estimation, exact tests, generalized linear models and quasi-likelihood tests were used to determine whether statistically significant differences existed between counts of one group of sequencing data to another through TMM normalized count matrices, controlling for sequencing depth between samples and datasets. Function and pathway enrichment analysis was performed with Metascape and enrichment of gene ontology (GO) was performed with Gene Set Enrichment Analysis (GSEA) [33,34].

### Statistical analysis

Baseline clinical and sociodemographic characteristics were compared between local recurrent and non-recurrent SCCA. Statistical comparisons with *p* < 0.05 were considered significant. Cancer staging was also summarized using the American Cancer Society’s SCCA stage criteria [35]. PCA (Principle Component Analysis) was used to examine the variance in overall expression patterns in each sample in a linear combination and the uncorrelated factors separated the orthogonal components. DEGs were considered upregulated or downregulated if (log_2_ fold change (log_2_FC))>2 and (log_2_FC) >-2, respectively and *p*-value < 0.05 were found between the recurrent and non-recurrent groups. EdgeR automatically adjusts p-values generated for each individual gene for multiple comparisons as shown in the user guide [32]. To examine various correlations of the transcripts, Volcano Plots and bubble plots were generated in R; Heatmaps, and bar graphs as well as other visualizations were generated using a combination of R, Metascape, Cytoscape, GSEA, and PathwayViz [33,34,36,37].

The study was approved by the Office of the Institutional Review Board of Human Use at UAB (IRB-300000131). It fully adhered to the Declaration of Helsinki.

## Results

Table 1 shows the demographics of the PLWH studied in the study. Study participants included 3 LR and 9 CR anal cancer patients, with male gender only. All participants underwent chemoradiation therapy as the initial primary treatment. Race/ethnicity, sexual risks (MSM), and HIV-related clinical indicators CD4 T counts at different time points were not statistically significant between recurrent and non-recurrent samples (Table 1).

**Table 1.** Baseline demographic and clinical factors compared between SCCA with and without recurrence.

	Non-recurrence *N* = 9	Recurrence *N* = 3	*p*- value
Race^b^			1.00
Black	4 (44.4)	1 (33.3)	
White	5 (55.6)	2 (66.7)	
Age at cancer diagnosis^c^	52.2 (4.2)	50.6 (5.1)	0.59
Sexual risk^†^			1.00
Heterosexual men	1 (11.11)	0 (0)	
MSM Unknown	6 (66.7) 2 (22.22)	3 (100) 0 (0)	
Immediate CD4 (cells/mm^3^) before AC^a^	265 (169–318)	415 (27–648)	0.58
Median CD4 (cells/mm^3^)^a^	300 (188–450)	282 (74–648)	1.00
Nadir CD4 (cells/mm^3^)^a^	102 (70–162)	169 (26–648)	0.40
cART^a^	9 (100)	3 (100)	--
Years since sample collected (years)^c^	8.3 (2.9)	7.2 (2.3)	0.604

^a^Variables were reported as median counts and 25%–75% interquartile range (IQR).

^b^Variables were reported as counts and percentage (%).

^c^Variables were reported as mean and standard deviation (SD).

### Cancer staging

None of the three recurrent SCCA were diagnosed with the same stage (TisN0M0, T1N0M0, and T4N0M0). Out of the 9 non-recurrent patients, 4 were T2N0M0 (44.4%), 2 were T1N0M0 (22.2%), 1 of each of TisN0M0, T3N0M0 and T4N0M0.

### Principal component analysis (PCA)

**Figure 1.**
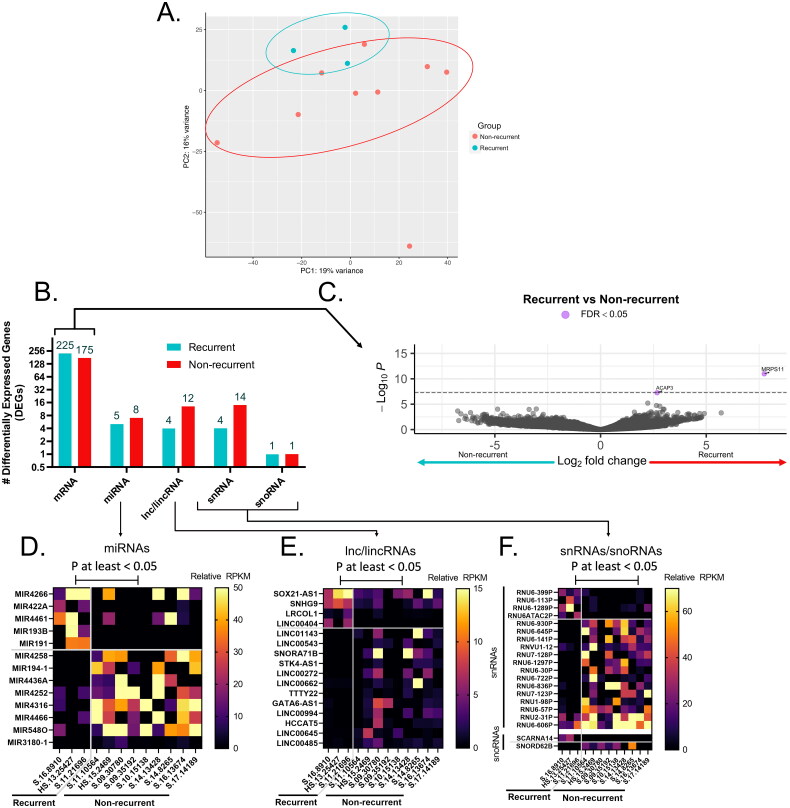
Transcriptional landscape of recurrent anal cancer. (A) Principle component analysis (PCA) of counts matrix corresponding to all non-recurrent or recurrent anal cancer isolates colored by group and clustered by top 2 principle components accounting for 16 and 19% of total variance. (B) Bar graph of number of total differentially expressed genes (DEGs) identified as upregulated and significantly different (*p* < 0.05) in either recurrent or non-recurrent isolates as mRNA, miRNA, lnc/lincRNA, snRNA, or snoRNA. All mRNA DEGs are represented as a (C) volcano plot while ncRNAs such as (D) miRNAs, (E) lnc/lincRNAs, and (F) snRNA/snoRNAs are represented as heatmaps. Only those mRNA DEGs which were identified as significantly different between groups by a FDR < 0.05 were highlighted in purple in (C).

Following gene-expression normalization, initial PCA indicated that a subset of all except one non-recurrent clustered and another subset of three recurrent clustered (Figure 1 A). However, a subsequent permutation analysis of variance analysis (PERMANOVA) confirmed that recurrence was not a significant factor governing the overall variance of samples within the PCA (*p* > 0.05).

#### Identification of DEGs between recurrent and non-recurrent samples

There were 449 DEGs (390 mRNA, 12 miRNA, 17 lincRNA and 18 snRNA) observed when comparing the recurrent to the non-recurrent group (Figures 1(B)). Two genes, *ACAP3* and *MRPS11* passed the false discovery rate (FDR) <0.05. Volcano plot (Figure 1(C)), indicated all the differentially expressed mRNAs that were statistically significant in the two groups. Heatmaps of specific miRNAs, lincRNAs and snRNAs that were differentially expressed are shown in Figures 1(D, E, F), respectively.

#### GO Functional annotation for DEGs using Metascape

**Figure 2.**
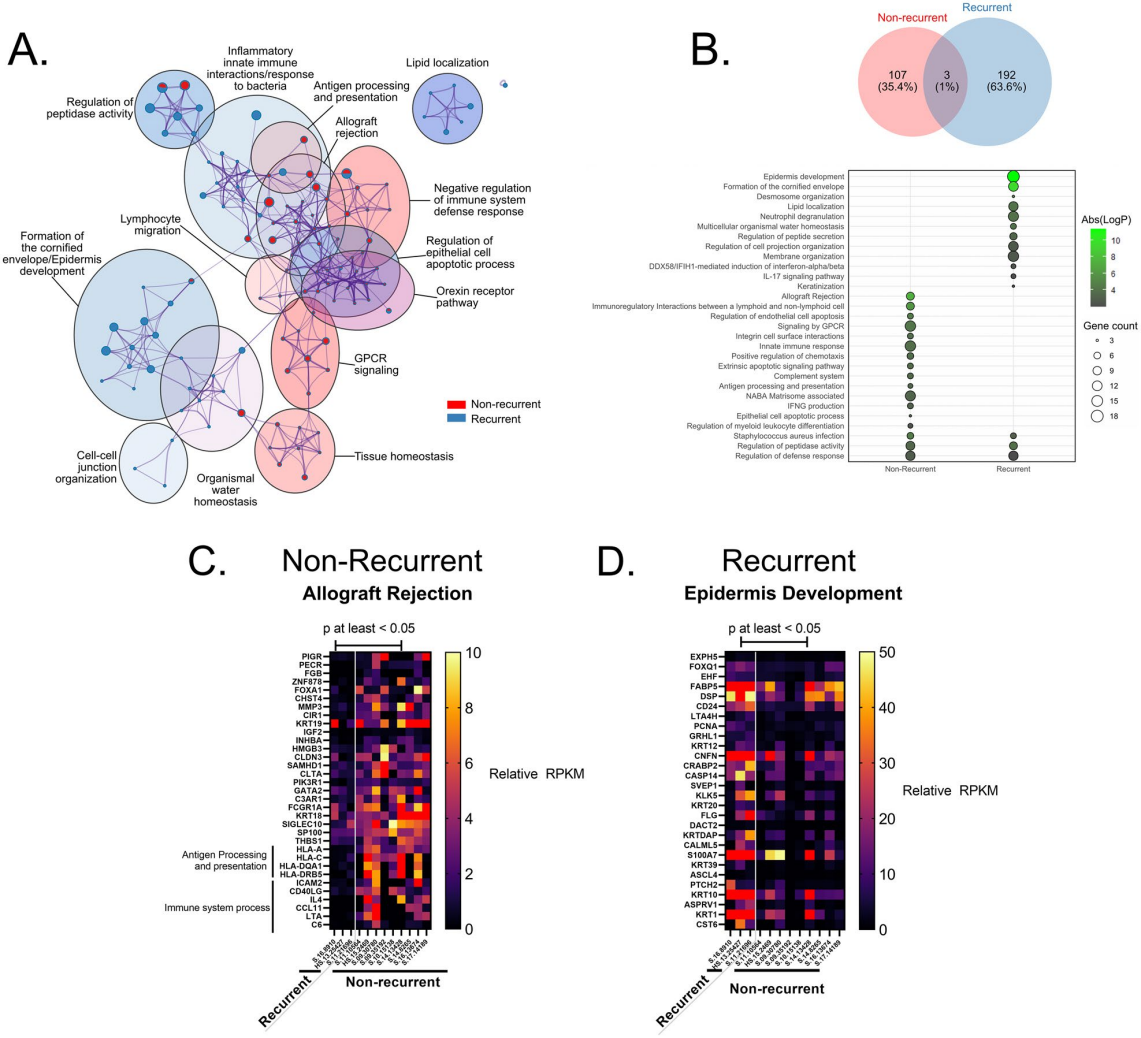
Gene ontology (GO) enrichment for DEGs upregulated in recurrent or non-recurrent anal cancer isolates. (A) Metascape enrichment bubblemap of GO terms describing DEGs in each group, with relative number of DEGs from each group contributing to each term/node represented as individual pie charts, colored by membership. (B) Number of GO terms for DEGs in each group represented as venn diagram and most relevant biological processes represented as a bubbleplot colored by Abs(log(p-value)) and size constrained by number of DEGs contributing to term and ordered by Abs(logP). Representative heatmaps of DEGs contributing to the GO terms with the highest enrichment score for each group are shown for (C) Non-recurrent (Allograft Rejection) and (D) recurrent groups (Epidermis Development), colored by reads per kilobase exon per million mapped reads (RPKM) expression value for each DEG.

Functional enrichment analysis with Metascape found that DEGs between recurrent and non-recurrent were significantly enriched in antigen processing and presentation (–logP = 3.0), allograft rejection (–logP = 7.31), *GPCR* signaling (–logP = 4.21), and *IFNG* production (–logP = 3.76) (Figure 2(A)).

#### Enrichment analysis in GSEA

Figure 2(B) shows results from GSEA in recurrent and non-recurrent SCCA and the number of genes in each and the –logP-value. Of all GO terms, only 3 were shared between groups, the regulation of peptidase activity (–logP = 3.99 or 3.48 in recurrent or non-recurrent tissues respectively), defense response (–log *p* = 2.01 or 2.85 in recurrent or non-recurrent tissues respectively), and Staphylococcus aureus infection (–logP = 3.11 or 4.69 in recurrent or non-recurrent tissues respectively), though the specific genes enriching to these terms were not (Figure 2(B)). A number of genes involved in ‘allograft rejection’ also contributed to the ‘immune system process’ term, as the same immunologic processes involved in rejecting non-self allografts may overlap with cancer rejection (Figure 2(C)) [38]. DEGs enriched in the recurrent tissues were mainly involved in epidermis development (–logP = 9.54) (Figure 2(D)). In the ‘epidermis development’ term, a number of keratin genes were included such as *KRT1, KRT10, KRT12*, and *KRT20,* as well as hedgehog signaling pathway transmembrane receptor gene *PTCHptch2* (Figure 2(D)).

## Discussion

Chemoradiation therapy (CRT) has emerged as the standard of care for anal cancer; however, data have shown treatment is ineffective in up to 40% of the treated patients, with recurrence in the same anatomical sites in 10–14% of patients. Given the significant gap in knowledge as to why treatment response differs, our study revealed many DEGS (coding and non-coding transcriptomics) that could potentially: a) help understand the biological mechanism; and b) serve as clinical biomarkers for recurrence of anal cancer post-therapy. Overall, we observed signatures of productive immune responses or epidermal development in non-recurrent or recurrent anal cancer isolates respectively.

Very little has been published regarding the two genes (*ACAP3* and *MRPS11),* which passed the FDR< 0.05 in our study. A previous study suggested that downregulated *ACAP3* was associated with ferroptosis, a type of cell death, in thyroid papillary carcinoma and implied a novel direction for cancer therapy [39]. Another study found extremely down-regulated *ACAP3* in patients with late-stage liver cancer compared to the early-stage [40]. *MRPS11* appeared to be a robust prognostic indicator in uveal melanoma; up-regulated *MRPS11* was associated with worse overall survival [41]. Therefore, upregulation of these two genes may serve as a biomarker of likely recurrence.

Those genes contributing to ‘defense response’ and ‘peptidase activity’ terms in recurrent tissues were mainly composed of a number of genes previously associated with cancer such as *S100A8, S100A9, TANK*, or *ADORA2B* [42–45]. Conversely, there were a number of productive immune genes contributing to ‘defense response’ in non-recurrent anal cancer isolates such as *IL4, HLA-A, CD200R1,* and *CD96. CD96* is commonly expressed on the surface of CD8+ T cells and NK cells and has been implicated as an immune checkpoint inhibitor capable of suppressing IFNG production as well as cytotoxicity and worsening patient prognosis in cancer [46,47]. For the non-recurrent isolates a number of integrins (*ICAM2*) and collagen proteins (*COL4A3, COL6A3*) contributed to the ‘regulation of peptidase activity’ term. Additionally, human leukocyte antigen (HLA) components from both MHC class Ia and II essential to antigen presentation and presentation were upregulated only in the non-recurrent samples including *HLA-A*, *HLA-C* and MHC-II genes such as *HLA-DRB5* and *HLA-DQA1*. HLA class I molecules are critical to the anti-cancer immune response, as has been shown previously, mediated primarily through recognition and lysis by cytotoxic T lymphocytes (CTLs) [48]. Previous meta-analysis reported that patients with overexpressed HLA class I molecules contributed to better overall survival. It may imply that a high expression level of HLA-A and HLA-C in initial non-recurrent anal cancer isolates may thereby result in better chances of tumor-free survival.

Of the miRNAs differentially expressed (Figure 1D), miR-4316 seems to indicate some biological significance in other cancers. It is significantly downregulated in gastric tumor tissues and cell lines compared to healthy tissues [49]. The study suggested that miR-4316 inhibited tumor proliferation and migration by repressing vascular endothelial growth factor A (VEGF-A) [49]. Downregulation of miR-4316 in bladder cancer tissues elevates expression of *ZBTB2* [50]. *ZBTB2* promotes tumorigenesis by repressing p21 and facilitating *PDK4* transcription [50]. In breast cancer, miR-4316 is inversely associated with an over-expressed circular RNA, circMYO9B in tumor tissues [51]. In colorectal cancer, miR-4316 was over-expressed compared to healthy tissues, suggesting it could be a potential biomarker for early colorectal cancer diagnosis [52].

Similarly to the lincRNAs differentially expressed in our study, SNORA71B and -SOX21-AS1 have been indicated in the pathogenesis of other cancers. LincRNA-SNORA71B promotes the migration of breast cancer cells across the blood-brain barrier *via* epithelial-mesenchymal transition (EMT), leading to brain metastasis from initial breast cancer [53,54]. In prostate cancer, the knockdown of SNORA71B reduces tumor cell proliferation, invasion, migration, and EMT [55]. *SOX21-AS1* plays a pivotal role in multiple cancers, including, lung, cervical, breast, oral, and colorectal cancers [56–59] and is involved in increased tumor proliferation, migration, and invasion in tumor tissues [56–59]. The lincRNA SOX21-AS1, as well as *SOX21, SOX2*, and *PIK3CA* (encoding a subunit of PI3K), were found to be highly expressed in the recurrent tissues of this study (Figure 1E). A number of studies showed that mutations within *PIK3CA* gene were found in multiple HPV-induced squamous cell cancers, such as head and neck cancers, cervical, and anal cancers [18–23]. SOX21-AS1’s target is SOX21, which regulates *SOX2* and ultimately the *PI3K* pathway [60]. *PI3K* and *SOX2* have previously been identified as regulators of the ‘stemmness’ of related head and neck squamous cancer stem cells and may thereby be acting to promote SCCA recurrence through a similar mechanism [61].

Non-recurrent tissues, representing those neoplasms that were isolated before any recurrence, may be seen as the ‘initial’ onset of anal cancer. In comparison, therefore, those recurrent isolates represent a successive re-emergence of these tumors after initial removal and treatment, and therefore can be treated as those that were refractory to treatment. In these isolates, a number of genes were identified as upregulated over initial non-recurrent samples, mainly involved in epidermal development such as a number of keratin-coding genes (*KRT1, 10, 12, 20*), hedgehog signaling (*PTCH2*), and genes previously identified as contributing to the proliferation of a diverse array of other cancers (*S100A8, A9, TANK, ADORA2B*) [42–45, 62–64]. Many of these DEGs were also associated with water/lipid homeostasis, which is consistent with disruption of the epidermal barrier maintained by keratinocytes, melanocytes, and other epidermal stem cells typically responsible for protection against water loss [65].

Several limitations of the study were noted. In the study, we only analyzed tissue samples from 9 non-recurrent patients and 3 recurrent patients; thus, the power for statistical analysis is limited; however, it is one of the first studies to compare the complete transcriptional profile of rare cancer based on treatment outcome in rare cancer. While the current findings need to be replicated and validated in a larger patient population, this study provides some preliminary insights into the biology of SCCA that can be followed up in the future. The staging of SCCA is not uniform in all the patients; however, there was heterogeneity in both groups and no differences statistically, so no major bias is expected in the analysis due to staging status. In addition, the study was based on EHR data from one hospital, which means other clinical data of the patients, such as treatment or outcomes outside of UAB healthcare system may be missed. However, the study participants included in the study were diagnosed, treated, and followed up at the same UAB healthcare system, reducing some of these limitations in this study.

Overall, this study suggests that a complex immune-regulatory network may be acting within initial non-recurrent anal cancer isolates which is disrupted upon recurrence. A number of productive immune signatures were identified as being upregulated within initial non-recurrent isolates alongside several immune checkpoint molecules that may play a role in the recurrence of subsequent anal cancer post-treatment. CD4+ T cell function and infiltration into the sequenced non-recurrent anal cancer isolates, specifically, seem to be the main immune mediators that disappear in recurrence. These results suggest that the absence of transcriptional signatures of productive CD4+ T cell activation and subsequent humoral responses are the main immune components that contribute to the recurrence of anal cancer. Additionally, this study has identified several potential transcriptional signatures that may prove useful as biomarkers to predict the recurrence of anal cancer post-resection of a tumor in patients.

## Data Availability

The data that support the findings of this study are available from the corresponding author SS, upon reasonable request.
